# Evolutionary Rescue Through Partly Heritable Phenotypic Variability

**DOI:** 10.1534/genetics.118.301758

**Published:** 2019-01-29

**Authors:** Oana Carja, Joshua B. Plotkin

**Affiliations:** * School of Computer Science, Carnegie Mellon University, Pittsburgh, PA 15213; † Department of Biology, University of Pennsylvania, Philadelphia, Pennsylvania 19304

**Keywords:** evolutionary rescue, time to extinction, bacterial persistence, stochastic switching, evolutionary bet-hedging, fluctuating environments, adaptation to environmental change

## Abstract

Environmental variation is commonplace, but unpredictable. Populations that encounter a deleterious environment can sometimes avoid extinction by rapid evolutionary adaptation. Phenotypic variability, whereby a single genotype can express multiple different phenotypes, might play an important role in rescuing such populations from extinction. This type of evolutionary bet-hedging need not confer a direct benefit to a single individual, but it may increase the chance of long-term survival of a lineage. Here, we develop a population genetic model to explore how partly heritable phenotypic variability influences the probability of evolutionary rescue and the mean duration of population persistence in changing environments. We find that the probability of population persistence depends nonmonotonically on the degree of phenotypic heritability between generations: some heritability can help avert extinction, but too much heritability removes any benefit of phenotypic variability. Partly heritable phenotypic variation is particularly advantageous when it extends the persistence time of a declining population and thereby increases the chance of rescue via beneficial mutations at linked loci. We discuss the implications of these results in the context of therapies designed to eradicate populations of pathogens or aberrant cellular lineages.

THE very first study in experimental evolution, led by W. D. Dallinger in the 1880s, attempted to demonstrate that populations can rapidly adapt to environmental change and that evolutionary rescue of a population from extinction depends on the rate of change (Dallinger 1887). Evolutionary rescue is the process by which a population is able to recover from environmental changes that would otherwise lead to a demographic decline and eventual extinction (Gomulkiewicz and Holt 1995; Bell and Gonzalez 2009; Gonzalez *et al.* 2013; Lindsey *et al.* 2013; Alexander *et al.* 2014; Carlson *et al.* 2014). Evolutionary responses that allow populations to adapt to change on a sufficiently fast timescale to prevent extinction have been the focus of considerable experimental and theoretical interest, across diverse biological systems. In the field of conservation biology, questions of rescue are framed around ensuring the survival of species in deteriorating global habitats (Palumbi 2001; Davis *et al.* 2005; Gonzalez *et al.* 2013). In contrast, in clinical contexts, the goal is the eradication of pathogens or harmful populations of cells (Gonzalez *et al.* 2013; Alexander *et al.* 2014). These two bodies of work share a common thread [as Maynard Smith (1989) emphasized, adaptation in threatened populations is not like ordinary adaptation, it is a race against extinction], yet each presents unique difficulties.

Here, we focus on the questions of population eradication that arise in medically relevant settings, where populations are often surprisingly resilient or recalcitrant to treatment due to evolutionary adaptation. In particular, we ask: how does heritable phenotypic variability alter the probability of evolutionary rescue? We study the evolutionary advantage of heritable phenotypic variability in populations of nonconstant size. We determine the probability of rescue and the mean time to extinction in changing environments, through both analytical approximation and Monte Carlo simulations of population genetic models.

We are motivated in asking this question by well-documented examples of phenotypic heterogeneity used as evolutionary bet-hedging strategies in volatile environments. Classic examples include: the bifurcation of a genotypically monomorphic population into two phenotypically distinct bistable subpopulations (Dubnau and Losick 2006); bacterial persistence (Kussell *et al.* 2005; Lewis 2010; Cohen *et al.* 2013; Sharma *et al.* 2015), whereby a genetically identical bacterial population survives periods of antibiotic stress by producing phenotypically heterogeneous subpopulations, some of which are drug-resistant (Lewis 2007); or quiescent phenotypes in cancer cell populations (Aguirre-Ghiso 2007; Sharma *et al.* 2010; Smith *et al.* 2016), which are transient phenotypic (epi)states protected from the action of drugs. These dormant phenotypic states confer the population with some degree of phenotypic heterogeneity, helping it withstand periods of environmental stress. Phenotypes may be partially heritable upon cellular division, so that the offspring cell can sometimes “remember” and express the phenotypic state of its parent, or sometimes switch between phenotypic states at rates that greatly exceed those of genetic mutations (Balaban *et al.* 2004; Van den Bergh *et al.* 2016). Partial phenotypic inheritance through epigenetic mechanisms can lead to faster rates of adaptation and environmental tracking than genetic mutations alone. Even though persisters in such populations rely on a nongenetic form of inheritance, the rate of “phenotypic mutation” (also called “epimutation”) is itself likely under genetic control (Levin and Rozen 2006).

Epigenetic bet-hedging strategies that use dynamic regulation of phenotypic variability can allow a population to persist and escape extinction, until more permanent genetic strategies arise. Many studies have addressed questions of genetic responses in evolutionary rescue (Orr and Unckless 2008, 2014; Lindsey *et al.* 2013; Martin *et al.* 2013; Alexander *et al.* 2014; Uecker *et al.* 2014; Uecker and Hermisson 2015; Wilson *et al.* 2017). Less attention has been given to the potential impact of phenotypic variability and of stochastic switching on evolutionary rescue (Chevin *et al.* 2013; Ashander *et al.* 2016). A recent study integrated stochastic demography with quantitative genetic theory in a model with evolving plasticity to explore the probability of rescue from a single, abrupt change in the environmental conditions (Ashander *et al.* 2016). Evolving plasticity was shown to facilitate evolutionary rescue unless the new environment was unpredictable.

Epigenetic plasticity, as studied in Ashander *et al.* (2016), can cause phenotypes to differ widely within a lineage, whereas purely genetically encoded phenotypes only allow offspring phenotypically similar to the parents. The type of phenotypic variability we explore here can produce phenotypic heterogeneity with familial correlations intermediate to these two extremes, as observed in the contributions of DNA methylation variation to the heritability of phenotypes in *Arabidopsis thaliana* for example (Johannes *et al.* 2009; Carja *et al.* 2014a; Carja and Plotkin 2017). This type of partly heritable phenotype is commonplace across biological systems and its role in evolutionary rescue is yet to be understood.

We explore the evolutionary fate of a population that experiences either one sudden shift in the environmental regime or many periodic changes in the environment. In the case of a single abrupt environmental change, we study the probability of rescue when one mutant allele permits the expression of multiple phenotypic states. We imagine these phenotypic states as partially heritable, so that the phenotype expressed by an individual will be inherited by the offspring with some probability, *p*. We call this probability *p* the phenotypic memory (Carja and Plotkin 2017). We are especially interested in the long-term fate of the population as a function of the variance in expressible phenotypes that the mutant allele confers, and also as a function of the amount of phenotypic memory between generations.

Our paper starts by specifying a mathematical model, based on birth–death processes, for populations subject to environmental change and to the introduction of a mutant allele that permits a range of expressible, partly heritable phenotypes. We pose our research question in terms of analyzing the long-term probability of extinction of such a population. We show that after one abrupt environmental change, the probability of evolutionary rescue is significantly increased when a new phenotypically variable allele is introduced in the population, and that this increase critically depends on the phenotypic memory of individuals expressing the variable allele. When the population experiences multiple environmental changes, the mean time to population extinction also increases for phenotypically heterogeneous populations (*i.e.*, population persistence increases) and this increase depends nonmonotonically on the phenotypic memory of the mutant allele, *p*. We also show that partly heritable phenotypic variation can be particularly advantageous for populations under threat of extinction when it extends the persistence time of the declining population and thereby increases the chance of rescue via beneficial, resistance mutations at linked loci. We provide a simple intuition for the complex dependence of evolutionary rescue on the degree of phenotypic memory, and we discuss the implications of our results for the eradication of evolving populations in medical contexts.

## Methods

We use a continuous-time birth–death model to describe changes in allele numbers in a finite population of changing size *N*, with carrying capacity *K*. Each individual’s genotype is defined by a single biallelic locus A/a, which controls its phenotype. The *A* allele encodes a fixed phenotypic value, whereas individuals with the *a* allele may express a wider range of phenotypes, drawn from a fixed distribution. Here, we analytically study the case where the *a* allele has access to two different phenotypic values. By simulation, we also explore cases where the *a* allele has access to more than two phenotypic states. The phenotypic values available to the *a* allele are chosen such that both alleles *A* and *a* have the same expected fitness, so that the only difference between them is the possibility of (partly heritable) phenotypic variability. We analyze the probability of rescue as a function of the phenotypic variance and the phenotypic memory associated with the *a* allele.

We study two sets of questions related to phenotypic variability and persistence. In the first set of questions, the population, assumed to be initially fixed for the wild-type allele *A*, experiences a single abrupt change in the environmental regime at time t=0. This environmental shift is expected to lead to a demographic decline in the population, meaning that death rates exceed birth rates for allele *A*. We ask what is the probability of rescue when there is a positive mutation rate to the phenotypically variable allele *a*, which can provide an adaptive benefit. In this situation, there is a race between extinction of the population, and the establishment of a phenotypically variable *a* lineage. We also ask, what is the probability of evolutionary rescue from standing genetic variation; that is, whether the *a* allele is already present in the population at some frequency at time t =0. We study how the probability of rescue depends upon parameters such as the population size at time t = 0, the phenotypic variance of the *a* allele, the phenotypic memory of the *a* allele, and either the mutation rate toward *a* or the initial frequency of *a*.

In the second set of studies, we assume a population otherwise fixed on the nonvariable *A* allele with one phenotypically variable *a* allele introduced at time t=0, but here we assume multiple epochs of environmental changes, occurring periodically. The question of persistence is framed in terms of the mean time to extinction of the population, as a function of the environmental period, the phenotypic variance, and the phenotypic memory available to the *a* allele. In this case, the mapping from phenotype to fitness depends on the environmental regime, and it is chosen so that both alleles have the same expected fitness across environments.

### Evolutionary rescue from a single environmental change

**Figure 1:**
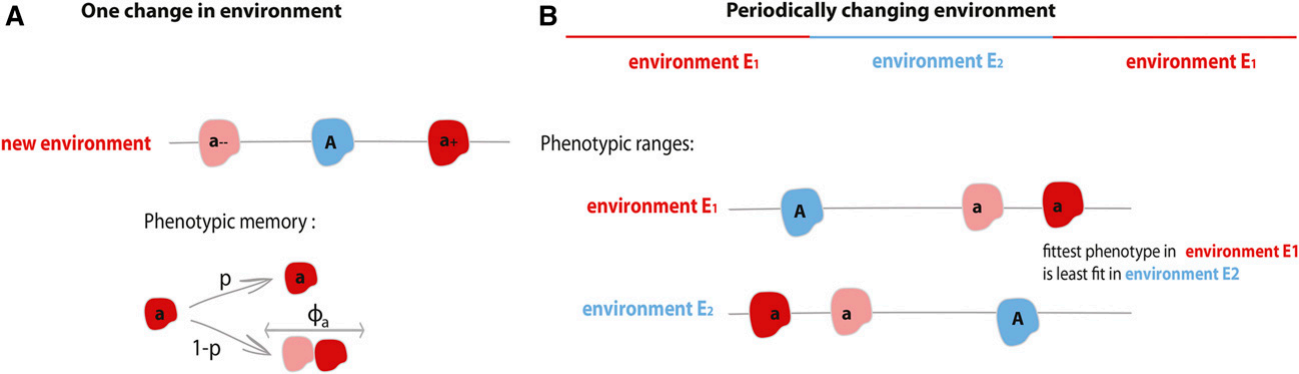
Illustration of the two versions of the model [figure adapted from Carja and Plotkin (2017)]. We study both one abrupt change in the environment (A) and periodic changes in selective pressure (B). In both cases, when an individual *a* gives birth, with probability *p* (the probability of phenotypic memory) its offspring inherits the phenotype of its parent, and with probability 1−p the offspring’s phenotype is resampled from the phenotypic distribution.

The one abrupt environmental change model studies how a novel allele that can express multiple phenotypes might alter the probability of evolutionary rescue of a population otherwise headed toward extinction. We describe the population using a continuous-time birth–death model. We assume the death rates are density-independent, and we study both density-independent birth rates and density-dependent ones (see the Supplemental Material). For the density-independent model presented here, individuals of the wild-type *A* and mutant type *a* each give birth and die according to the following *per capita* rates:
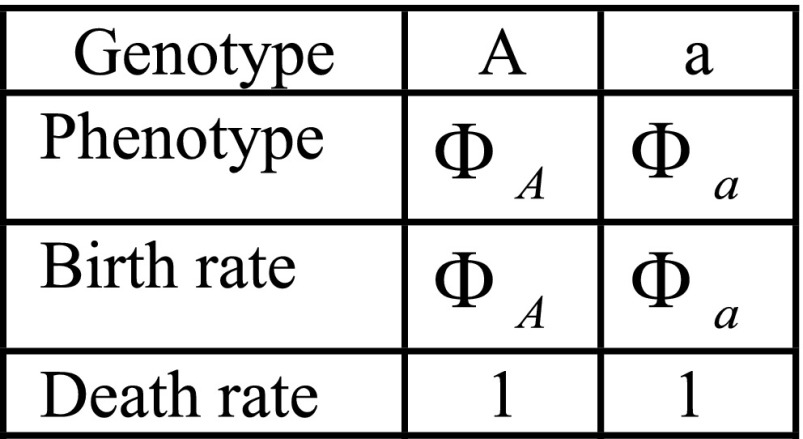


Here, $\Phi_{A}$ is a fixed number and $\Phi_{a}$ is a discrete random variable that can take two values, $\Phi_{a-}$ and $\Phi_{a+}$. We also denote the current population size by *N* and the carrying capacity by *K*. The death rates of the two alleles are both assumed to be in unity. The values $\Phi_{A}$ and $\Phi_{a}$ are constrained so that the two alleles have the same mean fitness: $\Phi_{A}=E\left(\Phi_{a}\right)$. We study fitness functions with equal means so that we can focus our analysis on the effect of variance in phenotypes expressed by allele *a*, Var$\left(\Phi_{a}\right)$, and not on any mean fitness effects. An illustration of this model is presented in Figure 1A.

In our analysis of this model, we initiate the population in a regime where the wild-type allele has a higher *per capita* death rate than its birth rate, so that a wild-type population is expected to become extinct fairly quickly. We analyze the conditions under which the mutant allele *a* (arising either by mutation or from standing variation at time t=0) will rescue the populations from extinction. We compare this analysis with Monte Carlo simulations, in which we record the proportion of replicate simulations in which rescue occurs.

### Population persistence in periodically changing environments

In our analysis of periodic environmental changes, we assume that the population experiences two different environments, $E_{1}$ and $E_{2}$, which alternate every *n* generations, so that the environmental period is 2n generations. We assume that one environment is more favorable to one allele and the other environment is more favorable to the other allele; that is, we study a model where the phenotypically variable allele *a* has lower expected fitness than the wild-type allele in one of the environmental regimes (for example the “no antibiotic” regime) and it has higher expected fitness than the wild-type in the other regime (for example with antibiotic pressure) (Carja and Plotkin 2017).

We choose phenotypic ranges so that the mean fitness expressed by each of the two genotypes are equal across the two environmental regimes. This setup allows us to focus on the evolutionary advantage of the phenotypic variance of *a*, Var$\left(\Phi_{a}\right)$, and to study population persistence without any effect of a difference in mean fitness.

Individuals of the wild-type *A* and mutant type *a* each give birth and die according to the following *per capita* rates:
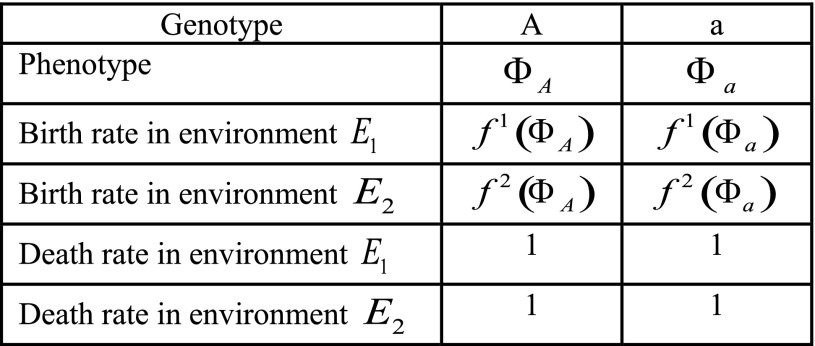


where $\Phi_{a}$ denotes a random variable and $\Phi_{A}$ is a fixed value. The functions $f^{i}:\mathbb{R}\to \mathbb{R}$ (i∈{1,2}) map phenotype to birth rate in each of the two environments, and $f^{1}$ is the identity function. We assume that both alleles have the same mean fitness in their optimal environment and the same mean fitness in their unpreferred environment: $E\left(f^{1}\left(\Phi_{A}\right)\right)=E\left(f^{2}\left(\Phi_{a}\right)\right)$ and $E\left(f^{2}\left(\Phi_{A}\right)\right)=E\left(f^{1}\left(\Phi_{a}\right)\right)$. This condition also ensures that the average of two alleles’ mean fitnesses, which we denote $M=\frac{E\left(f^{1}\left(\Phi_{A}\right)\right)+E\left(f^{1}\left(\Phi_{a}\right)\right)}{2}=\frac{E\left(f^{2}\left(\Phi_{A}\right)\right)+E\left(f^{2}\left(\Phi_{a}\right)\right)}{2}$, is the same in both environments. The function $f^{2}$ is defined as a reflection of $f^{1}$ around *M*: $f^{2}\left(x\right)=2M-f^{1}\left(x\right)$. As a result, the variance in fitness of allele *a* with a randomly drawn phenotype is the same in both environments: $\text{Var}\left(f^{1}\left(\Phi_{a}\right)\right)=\text{Var}\left(f^{2}\left(\Phi_{a}\right)\right)=\text{Var}\left(\Phi_{a}\right)$ (Carja and Plotkin 2017). For our analytical treatment, we assume that the *a* allele has access to only two phenotypic values, as depicted in Figure 1B, but Supplemental Material, Figures S8 and S9 show similar results in simulations of a model with more than two phenotypic states associated with allele *a*.

We study the possible long-term advantage of heritable phenotypic variability by analyzing how the introduction of the *a* allele into an otherwise nonvariable population (*A*) changes the population’s probability of evolutionary rescue. We quantify how the probability of rescue depends on environmental factors, such as the environmental period 2n; on demographic factors, such as the carrying capacity *K* if there is density dependence; and on molecular factors, such as the variance in phenotypes that can be expressed by allele *a*, Var$\left(\Phi_{a}\right)$, and the degree of phenotypic memory, *p*. We derive analytic approximations in the case of one environmental change and we determine the mean times to extinction in changing environments by Monte Carlo simulations (Gillespie 1976), using an ensemble of at least 10,000 replicate populations.

### Evolutionary rescue from a resistance mutation at a linked second locus

To explore the possibility that phenotypic variability allows populations to persist longer until more permanent genetic strategies can be found, we also study a model where each individual in the population is also defined by a second, linked locus, *R*, at which a resistance mutation can appear at a rate smaller than the mutation rate at the A/a locus. We compare the probability of evolutionary rescue due to a resistance mutation in populations fixed for the nonvariable allele *A* *vs.* populations that allow the phenotypically variable *a* allele, and focus on the case in which the *a* allele by itself is unable to save the population from extinction (the effective birth rate of *a* is smaller than its death rate, similar to the *A* allele). The birth rate of an individual carrying the resistance *R* allele is assumed to be independent of the genotype present at the A/a locus.

We simulate the birth–death process in continuous time as follows. We sample the waiting time for an event from an exponential distribution with a rate parameter equal to the sum of all possible rates beginning at time zero; we then randomly assign a specific event according to the relative probabilities of occurrence of each event type (birth or death events) and update the population status, time, and all event rates. If the event implemented is a birth, we then determine the phenotypic state of the offspring as follows. If the individual chosen to reproduce has genotype *A*, then the phenotypic state of the offspring always equals its parent’s (fixed) phenotypic value. However, for a reproducing individual with the *a* allele there exists a probability of phenotypic memory, denoted by the parameter *p*, between parent and offspring: with probability *p* the offspring retains the phenotypic state of its parent, and with probability 1−p the offspring’s phenotype is redrawn independently from the random variable $\Phi_{a}$. Thus, individuals of type *a* can express a range of phenotypic values and their phenotype is partly heritable between generations (provided p>0). In the case of periodic environments, we implement environmental changes (and recalculate event rates) at deterministic times: *n*, 2n, and 3n, *etc*. Time is measured in units of an individual’s expected lifetime; that is, the death rate is set to unity for all individuals in all simulations.

### Data availability

The authors state that all data necessary for confirming the conclusions presented in the manuscript are represented fully within the manuscript. Supplemental material available at Figshare: https://doi.org/10.25386/genetics.7639649.

## Results

### Evolutionary rescue from a novel mutation

We study the probability of evolutionary rescue after a single, abrupt environmental change that would lead to population extinction of the wild-type allele *A*. We assume that there is a constant rate of mutation from the *A* allele toward the *a* allele, μ. We obtain analytic approximations for the density-independent model and also discuss the density-dependent case in the Supplemental Material. Intuitively, the density-independent model applies in biological situations where the population is very far from carrying capacity *K* (such that the term $\left(1-\frac{N}{K}\right)$ is ∼1), while the density-dependent case applies for populations large enough at the time of environmental change such that $\left(1-\frac{N}{K}\right)$ cannot be ignored.

**Figure 2:**
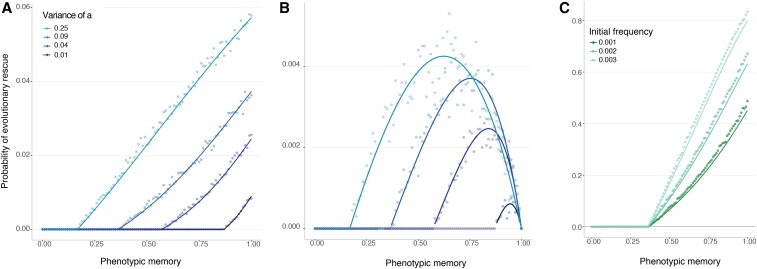
Probability of evolutionary rescue from a new mutation, density-independent birth. The lines represent the analytical approximations. The dots represent the ensemble average across 10,000 replicate Monte Carlo simulations. (A) Probability of rescue when *A* can only initially access the $a_{+}$ phenotype. (B) Probability of rescue when *A* can only initially access the $a_{-}$ phenotype. Here, K=5000, $N_{0}=1000$, $N_{0}\mu =0.01$, and $E\left(\Phi_{A}\right)=E\left(\Phi_{a}\right)=0.95$. (C) Probability of evolutionary rescue from standing genetic variation, density-independent birth. Here, $N_{0}=2500$, $E\left(\Phi_{A}\right)=E\left(\Phi_{a}\right)=0.95$, and Var$\left(\Phi_{a}\right)=0.09$.

The probability of establishment of the new mutant (and therefore the probability of population rescue) depends critically on the mutation rate to the *a* allele, and whether the first *a* individual is initially introduced with its beneficial or deleterious phenotype: that is, whether its birth rate is initially larger or smaller than its death rate. We first study the case where the *A* allele can only mutate to produce an *a* allele with the beneficial phenotype, denoted by $\Phi_{a_{+}}$. Biologically, this could be due to the presence or absence of an epigenetic marker that makes the deleterious $\Phi_{a_{-}}$ phenotype directly inaccessible from the *A* allele. The population will be rescued, by definition, if the *a* lineage manages to become established (Uecker and Hermisson 2011). As shown in Figure 2A, the chance of evolutionary rescue increases monotonically with the strength of phenotypic memory, *p*. This result makes intuitive sense: high-fitness variants of the *a* allele are preferentially transmitted to the next generation, and greater phenotypic memory *p* increases their propensity to maintain the high-fitness phenotype and become established in the population. Moreover, the probability of rescue is uniformly greater when the *a* allele can express a greater diversity of phenotypes, *i.e.*, for large Var$\left(\Phi_{a}\right)$ (Figure 2A), because the larger variance is associated with greater fitness of the $\Phi_{a_{+}}$ phenotype. In summary, when the variable allele is introduced with a beneficial phenotype, rescue is facilitated by increased phenotypic memory and by increased phenotypic variance of the phenotypically variable allele.

When the *a* allele is introduced with a deleterious phenotype $\Phi_{a_{-}}$, evolutionary rescue can still occur, because the phenotype of *a*-type individuals may change between generations. In this case, Figure 2B shows that the probability of evolutionary rescue depends nonmonotonically on the strength of phenotypic memory *p*. There is simple intuition for this result as well, which is informed by our mathematical analysis below. Intuitively, the probability of establishment in this case is the product of the probability that some *a*-type individual produces an offspring with the beneficial phenotype, $\Phi_{a_{+}}$, before the *a* lineage is lost, times the probability of establishment associated with such an individual with phenotype $\Phi_{a_{+}}$. Therefore, rescue is facilitated as the strength of phenotypic memory increases above zero (this effect is driven by the increase in the probability of rescue once an individual of phenotype $\Phi_{a_{+}}$ arises); but as the phenotypic memory increases further, toward one, the probability of rescue is reduced, because the entire *a* lineage will likely become extinct before producing any individual with a beneficial phenotype. Or, put differently, the *a* lineage needs sufficient variability to produce the correct phenotype, but not too much to avoid losing it after it has been produced.

To provide a clear analytical analysis of the intuitions described above, we first derive the probability of rescue, ${\mathbb{P}}_{r}\left(a_{+}\right)$, when there is recurrent mutation toward the beneficial phenotype $\Phi_{a_{+}}$. We first compute an effective selection coefficient of the entire *a* lineage, by assuming that the two phenotypes within the *a* lineage quickly reach epimutation–selection balance (Carja and Plotkin 2017). Given epimutation rate $u=\frac{1-p}{2}$ between the two phenotypes, the standard mutation–selection balance expression (where *x* refers to the frequency of the $\Phi_{a_{+}}$ phenotype),

$$\left(\Phi_{a_{-}}-\Phi_{a_{+}}\right)x^{2}-\left(\Phi_{a_{-}}-\Phi_{a_{+}}+u\left(\Phi_{a_{-}}+\Phi_{a_{+}}\right)\right)x+\Phi_{a_{-}}u=0,$$

implies that the equilibrium frequency of phenotype $\Phi_{a_{+}}$ within the *a* lineage is given by $f_{a_{+}}$:

$$f_{a_{+}}=\frac{\Phi_{a_{+}}-\Phi_{a_{-}}-u\Phi_{a_{-}}-u\Phi_{a_{+}}}{2\left(\Phi_{a_{+}}-\Phi_{a_{-}}\right)}+\frac{\sqrt{4\Phi_{a_{-}}u\left(\Phi_{a_{+}}-\Phi_{a_{-}}\right)+{\left(\Phi_{a_{-}}-\Phi_{a_{+}}+u\Phi_{a_{+}}+u\Phi_{a_{-}}\right)}^{2}}}{2\left(\Phi_{a_{+}}-\Phi_{a_{-}}\right)}$$ (1)

The effective birth rate of the *a* lineage is

$$s_{a}=\Phi_{a_{-}}\left(1-f_{a_{+}}\right)+\Phi_{a_{+}}f_{a_{+}}.$$ (2)

Taking into account that $\Phi_{a_{-}}=\Phi_{A}-\sigma_{\Phi_{a}}$ and $\Phi_{a_{+}}=\Phi_{A}+\sigma_{\Phi_{a}}$, this birth rate can be rewritten as a function of the mean $E\left(\Phi_{a}\right)$ and the standard deviation $\sigma_{\Phi_{a}}$ of the two alleles:

$$f_{a_{+}}=\frac{2\sigma_{\Phi_{a}}-2E\left(\Phi_{a}\right)u}{4\sigma_{\Phi_{a}}}+\frac{\sqrt{{\left(2E\left(\Phi_{a}\right)u-2\sigma_{\Phi_{a}}\right)}^{2}-8\sigma_{\Phi_{a}}\left(\sigma_{\Phi_{a}}u-E\left(\Phi_{a}\right)u\right)}}{4\sigma_{\Phi_{a}}}$$ (3)

and

$$s_{a}=\left(E\left(\Phi_{a}\right)-\sigma_{\Phi_{a}}\right)\left(1-f_{a_{+}}\right)+\left(E\left(\Phi_{a}\right)+\sigma_{\Phi_{a}}\right)f_{a_{+}},$$ (4)

as in Carja and Plotkin (2017).

When the effective birth rate $s_{a}$ of the *a* lineage exceeds its death rate (unity), the probability of establishment of this new mutation arising at time τ in the population is given by

$${\mathbb{P}}_{est,a_{+}}\left(\tau \right)=\frac{2}{1+{\int^{\text{​}}}_{0}^{\infty }\left(s_{a}\left(1-\frac{N\left(t+\tau \right)}{K}\right)+1\right)\exp\left(-{\int^{\text{​}}}_{0}^{t}\left(s_{a}\left(1-\frac{N\left(t\prime +\tau \right)}{K}\right)-1\right)dt\prime \right)dt}$$ (5)

following Uecker and Hermisson (2011) and Wilson *et al.* (2017). Assuming that the mutant lineages have independent probabilities of establishment, and thus neglecting the mutant population size, N(t) can be approximated by the *A* allele population size, which, due to the density-independence in birth rate for the wild-type *A* (but see the density-dependent case in the Supplemental Material), can be deterministically approximated by $N\left(t\right)=N_{0}\exp\left(-\left(1-\left(1-\mu \right)\Phi_{A}\right)t\right)$, where $N_{0}=N(t=0)$. This expression takes into account mutations away from *A* at rate μ.

We can now derive the probability of evolutionary rescue from at least one adaptive mutant by modeling mutant establishments using a time-inhomogeneous Poisson process with intensity function $\mu N\left(t\right)\Phi_{A}P_{est,a_{+}}$. The probability of evolutionary rescue then becomes one minus the probability that no mutants establish:

$${\mathbb{P}}_{rescue,a_{+}}=1-\exp\left(-\int_{0}^{\infty }\mu N\left(t\right)\Phi_{A}{\mathbb{P}}_{est,a_{+}}\left(t\right)dt\right),$$ (6)

which simplifies to

$${\mathbb{P}}_{rescue,a_{+}}=1-e^{-\frac{\mu N_{0}\Phi_{A}\left(s_{a}-1\right)}{\left(1-\left(1-\mu \right)\Phi_{A}\right)s_{a}}}.$$ (7)

This analytic approximation is shown in Figure 2A alongside the results of Monte Carlo simulations. For comparison to simulation, we defined rescue as the population reaching carrying capacity *K*.

Conversely, when the *a* mutation is introduced with its deleterious phenotypic state, ${\text{Φ}}_{a_{-}}$, we first derive an approximation for the probability of establishment ${\mathbb{P}}_{est,a_{-}}\left(t\right)$ as the probability of at least one epimutation to ${\text{Φ}}_{a_{+}}$ before the loss of the *a* allele, followed by the establishment of this $a_{+}$ mutation:

$${\mathbb{P}}_{est,a_{-}}=1-e^{-{\int^{\text{​}}}_{0}^{\infty }u\Phi_{a_{-}}N_{a-}\left(t\right){\mathbb{P}}_{est,a_{+}}\left(t\right)dt},$$ (8)

according to epimutation viewed as a time-inhomogeneous Poisson process, where

$$N_{a-}\left(t\right)=e^{-\left(1-\left(1-u\right)\Phi_{a_{-}}\right)t}$$ (9)

represents the decline in the *a* population from a single individual with negative expected growth rate and mutation away from $a_{-}$ at rate *u* [see also Serra and Haccou (2007)]. Then, the analytic expression for the probability of rescue becomes

$${\mathbb{P}}_{rescue,a_{-}}=1-\exp\left(-{\int^{\text{​}}}_{0}^{\infty }\mu N\left(t\right)\Phi_{A}{\mathbb{P}}_{est,a_{-}}\left(t\right)dt\right),$$ (10)

which simplifies to

$${\mathbb{P}}_{rescue,a_{-}}=1-e^{-\mu N_{0}\frac{\Phi_{A}}{1-\left(1-\mu \right)\Phi_{A}}{\mathbb{P}}_{est,a_{-}}},$$ (11)

where

$${\mathbb{P}}_{est,a_{-}}=1-e^{-\frac{u\Phi_{a_{-}}\left(s_{a}-1\right)}{\left(1-\left(1-u\right)\Phi_{a_{-}}\right)s_{a}}}.$$ (12)

This analytical approximation is shown in Figure 2B, alongside the results of an ensemble of Monte Carlo simulations.

In the Supplemental Material, and Figures S1 and S2, we discuss and show both analytic and simulation results for the equivalent density-dependent model. Figure S10 extends the parameter regime studied, exploring different mean birth rates for the A/a alleles. As expected, the density-independent approximation for the rescue probability is similar to the full density-dependent treatment for systems starting far from carrying capacity (Figure S3). For the sake of simplicity, the analysis that follows (cases of rescue from standing genetic variation or rescue from a resistance mutation) assumes that birth and death rates are density-independent.

### Evolutionary rescue from standing genetic variation

Evolutionary rescue may alternatively occur from an adaptive variant that exists, at low frequency, at the time of the environmental shift that dooms the wild-type genotype to extinction.

To study this, we assume that at time t=0, the population already contains a number *q* of *a* alleles, and we study the probability of rescue from this standing genetic variation (Figure 2C). In this case, the *a* lineage is assumed to be at epimutation–selection balance between its two phenotypic forms at the time of the environmental change t=0. The epimutation–selection balance is computed using Equation 1 and the probability of rescue becomes

$${\mathbb{P}}_{rescue}=1-\exp\left(-\left(f_{a_{+}}q\frac{\left(s_{a}-1\right)}{s_{a}}+\left(1-f_{a_{+}}\right)q\left(1-\exp\left(-\frac{u\Phi_{a_{-}}\left(s_{a}-1\right)}{\left(1-\left(1-u\right)\Phi_{a_{-}}\right)s_{a}}\right)\right)\right)\right).$$ (13)

The result of this approximation, together with Monte Carlo simulations, is shown in Figure 2C. As the phenotypic memory *p* increases, the phenotypic epimutation toward $a_{-}$ decreases, and therefore the fraction of $a_{-}$ phenotypes initially present in the population decreases. Since the probabilities of establishment for the deleterious $a_{-}$ phenotypes are smaller than the ones for the $a_{+}$ phenotypes, the initial number of $a_{+}$ phenotypes is the main driver of the evolutionary dynamics.

### Evolutionary rescue from a novel resistance mutation at a secondary locus

We adapted our model to study the possibility that phenotypic variability might allow the population to persist until a more permanent genetic change, such as a resistance mutation, can establish.

**Figure 3:**
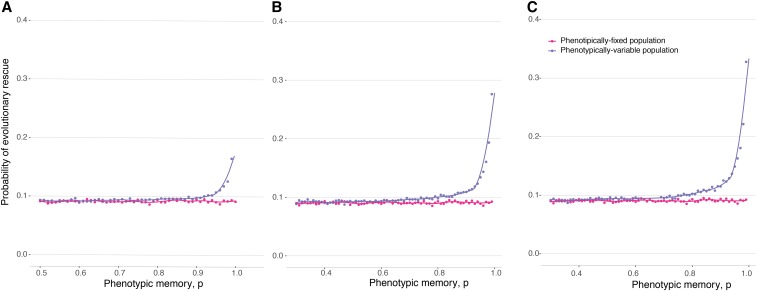
Probability of evolutionary rescue with a resistance locus. Here K=20000, $N_{0}=10000$, $E\left(\Phi_{\text{A}}\right)=E\left(\Phi_{a}\right)=0.95$, Var$\left(\Phi_{a}\right)=0.0025$, birth rate of *R* is 2, and mutation rate to R is $10^{-6}$. The dots represent results of a simulation, while the curves show cubic spline fits to the simulated data. (A) Mutation rate to *a* is 0.01. (B) Mutation rate to *a* is 0.05. (C) Mutation rate to *a* is 0.1.

We find that phenotypically heterogeneous populations have an advantage over populations composed of individuals of fixed phenotype (Figure 3 and Figures S4, S5, and S11). Even in the regime when the *a* allele cannot itself rescue the population from extinction, the probability of rescue from a resistance locus *R* can be significantly greater for a population with recurrent mutation to *a* (purple line) compared to a population fixed for the phenotypically invariant *A* allele (pink line). This effect holds only for relatively high mutation rates to *a*. The higher this mutation rate and the larger the fitness advantage of *R*, the wider the range of phenotypic memory for which this result holds. Since the probability of rescue when *a* and *R* are available to the population can be strictly greater than the probability of rescue from *R*, keeping in mind that the probability of rescue from *a* is zero, this result represents a form of epistatic interaction between the a/A locus and the *R* locus. The epistatic interaction arises because the *a* allele increases the expected time to extinction, even though it cannot alone rescue the population, and this additional persistence time increases the chance of acquiring a rescue mutation at the resistance locus.

### Population persistence in periodically changing environments

Phenotypic variability and phenotypic memory also influence population persistence in periodically changing environments, in addition to the case of a single environmental change that has been the subject of most work on evolutionary rescue. With a periodically changing environment, the question of persistence is conveniently framed in terms of the mean time before population extinction. Even in this more complicated setting, we once again observe a nonmonotonic effect of phenotypic memory, *p*, on population persistence: populations go extinct quickly for either small *p* or large *p*, whereas intermediate amounts of phenotypic memory can promote persistence for long periods of time.

**Figure 4 (A and B):**
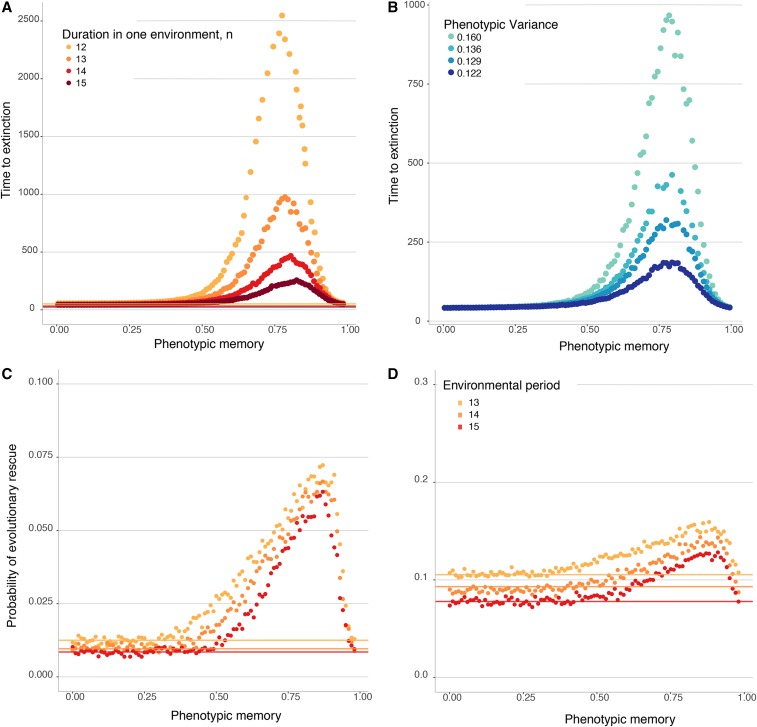
Population persistence in periodically changing environments. The dots represent the mean time before population extinction across 10,000 replicate simulations. All populations are initiated at N=500, with a carrying capacity K=10000, with a single-mutant genotype *a* drawn with a random phenotype introduced into one of the two random different environments. The two environments then cycle deterministically with each environmental epoch lasting *n* time units, where time is measured in units of the expected life span of an individual. (A) Mean time until population extinction for a range of different environmental periods, with $f^{1}\left(\Phi_{A}\right)=0.5$, $f^{2}\left(\Phi_{A}\right)=1.5$, and Var$\left(\Phi_{a}\right)=0.16$. The lines, each corresponding to a different value of *n*, show the mean time to extinction of a population comprised of only wild-type *A* alleles. (B) Mean time until population extinction for different amounts of phenotypic variability, Var$\left(\Phi_{a}\right)$, with a fixed environmental duration of n=13 units, $f^{1}\left(\Phi_{A}\right)=0.5$, and $f^{2}\left(\Phi_{A}\right)=1.5$. (C and D) Probability of evolutionary rescue in periodically changing environments from a resistance mutation. The dots represent the probability of evolutionary rescue for a phenotypically variable population, while the lines represent this probability for a phenotypically fixed population, across 10,000 replicate simulations. All populations are initiated at N=500, with a carrying capacity K=5000, with a single-mutant genotype *a* drawn with a random phenotype introduced into one of the two random different environments. The two environments then cycle deterministically with each environmental epoch lasting *n* time units (with *n* as in the legend), where time is measured in units of the expected life span of an individual. Here, the birth rate of the resistance allele *R* is 1.5, $f^{1}\left(\Phi_{A}\right)=0.5$, $f^{2}\left(\Phi_{A}\right)=1.5$, and Var$\left(\Phi_{a}\right)=0.16$. (C) Mutation rate to *R* is $10^{-6}$. (D) Mutation rate to *R* is $10^{-5}$.

Figure 4A shows the mean time to extinction as a function of the phenotypic memory for a range of environmental periods *n*. In all these cases, a population comprised of only the nonvariable wild-type allele *A* goes extinct quickly (compare with Figure S6), but populations initiated with a single copy of the phenotypically variable allele *a* have the potential to persist for very long times, especially for intermediate values of the phenotypic memory parameter *p*.

In our model of periodic environments, faster environmental changes are correlated with longer population persistence, even in the case of a phenotypically invariant wild-type population (Figure S6). This occurs because long stretches of the environmental regime deleterious to the *A* allele lead to population declines that the beneficial periods cannot replenish. It is particularly in faster-changing environments that phenotypic memory in a phenotypically variable allele *a* provides the largest advantage for population persistence, because it helps the high-fitness realizations of the *a* allele remain high in fitness, which is essential for population persistence through environmental epochs deleterious to the wild-type *A* allele. The nonmonotonic dependence of persistence time on phenotypic memory also makes intuitive sense. On the one hand, it is beneficial for the *a* allele to have some phenotypic memory within each environment ($E_{1}$ or $E_{2}$), as this helps the high-fitness realizations of the allele with little effect on its low-fitness realizations. On the other hand, too much phenotypic memory is detrimental in the long-run, because once the environment shifts, the *a* lineage will be “stuck” with a deleterious phenotype. Moreover, regardless of the phenotypic memory, the duration of persistence always increases with the variance in phenotypes that *a* can express, Var$\left(\Phi_{a}\right)$ (Figure 4B); that is, the population can persist longer when the phenotypically variable allele has access to a larger phenotypic range.

Similarly to the case of a single change in environment, the phenotypically variable allele allows the population to persist for longer and thereby increases the chance that a resistance allele will arise and establish, rescuing the population from extinction (Figures 4C and 4D and Figure S7). The epistatic effect between a phenotypically plastic allele and a resistance allele observed in Figure 3 is also observed in periodically changing environments; while the *a* allele by itself cannot rescue the population from extinction, the probability of rescue for a population that has access to both *a* and *R* is significantly elevated (with a maximum for intermediate memory *p*) compared with a population fixed on *A* with access only to the resistance allele *R*.

### Multiple phenotypes

We also explore the probability of rescue and time to extinction for populations in which the *a* allele has access to more than two different phenotypes. In Figure S8 we show by simulation that, when the *a* allele has access to four or six different phenotypes, as long as the phenotypic variance of the *a* allele is kept constant, the rescue probabilities are almost the same as the case of a phenotypic random variable with two-point masses. The same holds for the time to extinction in periodically changing environments (Figure S8C). We also vary the number of phenotypes available to the *a* lineage while keeping its phenotypic range constant (increasing the number of phenotypes available with a constant phenotypic range decreases the phenotypic variance). The probability of evolutionary rescue decreases with the number of phenotypes in accordance with the accompanying decrease in the phenotypic variance (Figure S9).

## Discussion

Evolutionary adaptation occurring on the same timescale as demographic dynamics can have profound effects on the persistence of a population. The theory of evolutionary rescue provides a conceptual framework that links demography and evolution in finite populations of variable size (Orr and Unckless 2008, 2014; Lindsey *et al.* 2013; Alexander *et al.* 2014; Uecker *et al.* 2014; Uecker and Hermisson 2015; Bertram *et al.* 2016; Wilson *et al.* 2017). Populations experiencing a sudden change in selection pressures, or frequent and unpredictable environmental variation, may either genetically adapt or be unable to recover. These populations have a limited window of opportunity for individuals with phenotypic solutions advantageous in the novel environment to appear and establish. Genetic adaptation after abrupt environmental changes can prevent extinction under several different demographic scenarios, but such a mechanism of rescue is inherently limited by the genomic mutation rate.

It is widely recognized that phenotypic variation can serve as a response to environmental change (Botero *et al.* 2015; Tufto 2015). The goal of this study has been to develop a population genetic model to quantify the probability of evolutionary rescue and mean times to extinction for populations that can access an allele with increased, partly heritable phenotypic variation. We have studied the problem of evolutionary rescue both from a novel mutation or for preexisting standing variants. Our analytical results are restricted to the case in which the phenotypically variable lineage can access two different phenotypes, but we also show that the results hold in simulation for alleles with more than two phenotypic states. The model with exactly two phenotypic states can be easily reframed as a model of stochastic phenotypic switching. Here, the switching rate can be written as $\frac{1-p}{2}$, where *p* is the phenotypic memory described in this paper. This reframing helps place some of our results in the context of the larger literature on phenotypic bet-hedging that has been developed both for infinite and finite populations, and for environments fluctuating through time (Balaban *et al.* 2004; Kussell *et al.* 2005; Salathé *et al.* 2009; Carja *et al.* 2014a; Carja and Plotkin 2017).

Previous theoretical studies on the adaptive benefit of stochastic phenotypic variability have mostly analyzed the evolution of rates of phenotypic switching by modifier loci, and mostly in infinite populations (Salathé *et al.* 2009; Carja *et al.* 2014a). These studies have found that phenotypic switching rates should evolve in tune with the correlation between the environments of parent and offspring: when the environment fluctuates periodically between two states with different optimal phenotypes, the uninvadable switching rate between phenotypic states will evolve to approximately 1/*n*, where *n* is the number of generations between environmental changes (Ishii *et al.* 1989; Salathé *et al.* 2009). Further studies generalized these results to include both environmental and spatial fluctuations in selection (Carja *et al.* 2014a,b). We have previously explored the dynamics of phenotypic switching rates in finite populations of fixed size, where we found that the fixation probability of phenotypically variable alleles depends nonmonotonically on the probability of phenotypic inheritance between parent and offspring: probabilities of fixation are maximized at intermediate rates of phenotypic switching in fluctuating environments (Carja and Plotkin 2017).

Under periodic changes in selective pressures, we show that a phenotypically variable allele increases the time to extinction. Moreover, this increase is nonmonotonic: there exists an intermediate phenotypic switching rate that maximizes population persistence. The optimal rate of epimutation induces proportions of standing phenotypes in sync with the rate of environmental change, similar to the findings in Carja and Plotkin (2017). Partly heritable phenotypic variation can also rescue populations in decline following a single environmental change. We have derived approximations for the probabilities of rescue, both for a model of density-dependent selection as well as a simpler model of density-independent selection. While the two models lead to qualitatively similar evolutionary dynamics of the phenotypically plastic allele, the optimal memory rates are up to three times larger under density-independent selection compared to density-dependent selection.

The most striking result occurs when the allele providing phenotypic variation cannot rescue the population alone, but it can nevertheless facilitate rescue when linked to targets of beneficial mutations. Transient and variable phenotypes, which can be mediated by rapid transitions in the epigenome, may provide an additional, selectable layer of traits that enable populations to persist until the appearance of more permanent strategies, such as genetic resistance. In empirical settings, ranging from bacterial infections to latency in viral populations, or cellular neoplastic development, this form of epigenetic, partly heritable phenotypic heterogeneity has been shown to facilitate adaptation and persistence under changing selection pressures (Seger and Brockman 1987; Acar *et al.* 2008; Veening *et al.* 2008). Responses mediated by partially heritable phenotypic variability can occur on faster timescales than genetic responses, and they may play a critical role in the path toward long-term resistance eventually reinforced by genetic changes. Indeed, here we explored the fate of populations waiting for a resistance mutation, and we found that phenotypically variable populations have a higher probability of rescue via resistance than less-variable populations, both for the evolutionary rescue scenario as well as a scenario with periodically changing environments. One caveat is that these results hold only for very large rates of mutation from the wild-type to the persister phenotype *a*. We believe that future theoretical work of interest includes qualitative as well as quantitative explorations of a larger parameter space for this model of evolutionary rescue through initial persistence, followed by genetic resistance, as well as a rigorous exploration of the contribution of other evolutionary forces to the epistatic effects observed here.

Although our analysis does not describe the myriad of specific mechanisms that give rise to phenotypic variability, our model nonetheless provides qualitative and quantitative predictions that should hold broadly, and can help inform the design of therapies in clinical contexts where population eradication is desired. Indeed, many clinical examples of therapy failure are now known to be caused by phenotypic heterogeneity, persistence, or quiescent cellular states (Cohen *et al.* 2013; Deris *et al.* 2013).

By exploring the interplay between phenotypic memory and treatment period, our results suggest that two very different types of intervention will be effective. Both options stem from the fact that, unlike genetic changes, epigenetic or phenotypic changes are reversible. The existence of an intermediate phenotypic memory that maximizes the time to extinction suggests that effective interventions are treatments that disrupt the molecular memory to either extreme (p=0 or p=1). This would facilitate eradication by decreasing the chance of a phenotypically resistant type establishing before the population becomes extinct. Of course, further detailed predictive models, specialized to particular populations and drug actions, are needed to formulate optimal therapies across the plethora of diseases where transient phenotypic variability drives treatment failure; but we expect the lessons learned from simple models, concerning the complex effect of phenotypic memory on persistence, to hold generally.
